# Clinical outcome of myelodysplastic syndrome progressing on hypomethylating agents with evolving frontline therapies: continued challenges and unmet needs

**DOI:** 10.1038/s41408-022-00691-9

**Published:** 2022-06-24

**Authors:** Ahmad Ghorab, Aref Al-Kali, Michelle Elliot, Naseema Gangat, Hasan Alkhateeb, Mithun Shah, Yuanhang Liu, Cecilia Arana Yi, Hemant Murthy, Mohamed Kharfan-Dabaja, Ayalew Tefferi, Mrinal Patnaik, Mark Litzow, Talha Badar

**Affiliations:** 1 Division of Hematology and Oncology, Mayo Clinic, Jacksonville, FL USA; 2 Division of Hematology, Mayo Clinic, Rochester, MN USA; 3 Department of Quantitative Health Sciences, Mayo Clinic, Rochester, MN USA; 4 Division of Hematology and Oncology, Mayo Clinic, Phoenix, AZ USA

**Keywords:** Leukaemia, Myelodysplastic syndrome

## Introduction

Hypomethylating agents (HMA) are the standard treatment of patients with higher risk MDS with overall response rates in the range of 30–40% [1, 2]. A significant proportion of patients progressing after HMA therapy transform to AML. The majority of these patients carry adverse risk chromosomal/molecular features, are older, ineligible for intensive therapies, and therefore, have a poorer prognosis [3, 4]. While CPX-351 compared to 3 + 7 demonstrated improved outcome in AML patients with myelodysplastic related changes (MRC), there was no significant difference in overall survival (OS) in patients who received prior HMA therapy (5.7 vs 5.9 months) [5]. The azacitdine plus venetoclax combination compared to azacitdine alone demonstrated improve survival in patients with secondary AML, however in the pivotal study, previous receipt of any HMA, venetoclax, or chemotherapy for MDS was exclusionary [6]. We sought to explore outcome of MDS patients progressing after HMA therapy in the era of novel therapies utilizing real world data.

## Methods

This is a retrospective study that includes 71 MDS patients progressing on HMA therapy and treated at the Mayo Clinic. The study was conducted after institutional review board approval. Clinical details including baseline characteristics including cytogenetics (CG) and molecular data, treatment after HMA progression and clinical outcome was abstracted from electronic medical records of patients treated at Mayo Clinic between February 2015 and February 2021. Mutation analysis was performed on bone marrow specimens using a 42 gene targeted next generation sequencing (NGS) panel. Responses were assessed according to the International Working Group (IWG) response criteria for MDS and AML [7, 8]. Continuous variables were summarized as median (range), while categorical variables were reported as frequency (percentage). Predictors of treatment response were assessed by Chi-square or Fisher’s exact test for nominal data and Wilcoxon rank-sum test for continuous variables. Overall survival from the date of HMA progression till last follow up or death was evaluated by the Kaplan–Meier method with differences compared by the log-rank test. Cox proportional hazards regression models were used to find the univariate and multivariate predictors of overall survival. Multivariable models included all significant univariate predictors with *p* value < 0.1.

## Results

### Baseline characteristics

The baseline characteristics of patients at the time of progression after HMA therapy are summarized in Table 1. The median age was 67 years (range, 36–91). Twenty four percent (*n* = 17/71) of patients were diagnosed with therapy related MDS (t-MDS) and 48% (*n* = 34/71) of patients had complex CG. Six (8%), 13 (18%), 19 (27%) and 33 (46%) patients had IPSS-R low risk, intermediate risk, high risk and very high-risk disease at the initiation of hypomethylating therapy, respectively. The median number of cycles of HMA patients received prior to progression was 4 (range, 1–20) and time from MDS diagnosis to HMA progression was 10.9 months (range, 1.6–79.4). Five (7%) patients with prior HMA therapy, received allogeneic stem cell transplantation and had disease progression. Seven (10%) patients acquired complex CG at progression after HMA therapy. At the time of progression, 13 (18%), 10 (14%) and 48 (68%) patients had MDS with excess blast (MDS-EB1), MDS-EB2 and AML, respectively. Most commonly occurring mutations observed in ≥10% of patients were *TP53* (44%), *ASXL1* (21%), *RUNX1* (14%), *RAS* (13%) and *TET2* (11%) as summarized in Table 1. Supplemental Fig. 1 provides a heat map illustration of CG and mutation profile of patients at MDS diagnosis and at progression after HMA therapy.

**Table 1:** Baseline characteristics, treatment, and responses.

	No. (%), or Median [range]
Age, years	67 [36–91]
Male	44 (62)
**MDS IPSS-R at diagnosis**	
Low risk	6 (8)
Intermediate risk	13 (18)
High risk	19 (27)
Very high risk	33 (46)
Therapy-related MDS	17 (24)
BM blasts, %	52 [0-95]
Complex cytogenetics	34 (48)
High risk cytogenetics	40 (56)
**HMA prior to progression**	
Azacitidine	38 (53)
Decitabine	30 (42)
Azacitidine plus investigational agent	3 (4)
Number of HMA cycles	4 (1–20)
History of prior allogeneic stem cell transplantation	5 (7)
Time from MDS diagnosis to HMA progression (months)	10.9 [1.6-79.4]
**MDS IPSS-R at HMA progression (*n*** = **23)**	
Intermediate risk	5 (22)
High risk	3 (13)
Very High risk	15 (65)
**Disease status at progression**	
MDS-EB1	9 (14)
MDS-EB2	14 (18)
AML	48 (68)
**Mutations at HMA Progression**	
*TP53*	31 (44)
*ASXL1*	15 (21)
*RUNX1*	10 (14)
*RAS*	9 (13)
*TET2*	8 (11)
*SRSF2*	5 (7)
*BCOR*	5 (7)
*IDH1 or IDH2*	4 (6)
*U2AF1*	4 (6)
*EZH2*	4 (6)
*CBL*	3 (4)
*DNMT3A*	2 (3)
*STAG2*	2 (3)
*KDM6A*	2 (3)
*DDX41*	1 (1)
*SETB1*	1 (1)
*GATA2*	1 (1)

First-line therapy after progression on HMA/ complete response rate [number treated/CR-CRi-mCR rate (%)]	MDS-EB1 *n* = 10		MDS-EB2 *n* = 13		AML *n* = 48	
	TP53m *n* = 3	TP53 wt *n* = 6	TP53m *n* = 4	TP53 wt *n* = 10	TP53m *n* = 24	TP53 wt *n* = 24
Venetoclax-based therapy (combination with LDAC or HMA)	2/50%	3/0%	4/25%	7/43%	12/42%	14/71%
CPX-351	0	0	0	2/0%	4/0%	6/33%
Other low intensity therapies*	1/0%	4/25%	0	1/0%	1/50%	3/33%
Best supportive care	0	0	0	0	5/0%	0
Intensive chemotherapy (7 + 3 or High dose cytarabine based)	0	0	0	0	2/50%	1/100%

*MDS-EB* MDS with excess blast, *LDAC* low dose cytarabine, *HMA* hypomethylating agent, *m* mutated, *wt* wild type.

*Other low intensity chemotherapy (IDH1/IDH2 inhibitor, Gemtuzumab ozogamicin, ruxolitinib, alternate hypomethylating agent).

^a^5 AML patients did not received induction chemotherapy.

### Response

Among patients with MDS-EB1/MDS-EB2 (*n* = 23), 7 (30%) patients achieved complete remission (CR) and 2 (9%) had marrow CR. Among 42 AML patients who progressed after HMA therapy and received induction chemotherapy, 36% of patients achieved CR/CR with incomplete count recovery (i) (*n* = 8 CR, *n* = 7 CRi). Five (10%) out of 48 patients who progressed to AML, did not received induction chemotherapy due to poor performance and inadequate organ functions. Overall, 14 (20%) patients progressing on HMA successfully bridge to alloHCT. Apart from age ≥ 70 years (*p* = 0.02), there was no significant difference in baseline characteristics, mutation profile and treatment received on HMA progression among patients who achieved CR/CRi and received alloHCT versus no alloHCT (Supplementary Table 1).

We also evaluated determinants of complete response rate (CR + CRi + marrow CR) using baseline variables. Complex CG (24% vs. 76%; *p* = 0.04) and *TP53* mutation (19% vs. 81%; *p* = 0.04) predicted significantly inferior complete response rate (Supplementary Table 2). Whereas patients with *TET2* mutation had significantly higher complete response rate (75% vs. 25%; *p* = 0.01).

The proportion of patients who achieved complete response based on therapy at HMA progression were 46% (*n* = 19/41), 14% (*n* = 2/14), 67% (*n* = 2/3), 12.5% (*n* = 1/8) and 0% (*n* = 0/5) with venetoclax based combination, CPX-351, intensive chemotherapy, other low intensity therapies and best supportive care, respectively (*p* = 0.053). We also evaluated response rate (CR/CRi/mCR) with reference to different disease type, *TP53* mutation status and therapy. Among *TP53* mutated MDS-EB1 (*n* = 3), MDS-EB2 (*n* = 4) and AML (*n* = 24) patients; 2/3 (66%), 4/4 (100%) and 12/24 (50%) received venetoclax based therapy in MDS-EB1, MDS-EB2 and AML group with CR/CRi rate 50%, 25% and 42%, respectively (Table 1). Four (16%) AML patient with TP53 mutation, received CPX-351 and none achieved complete response.

### Survival

**Fig. 1:**
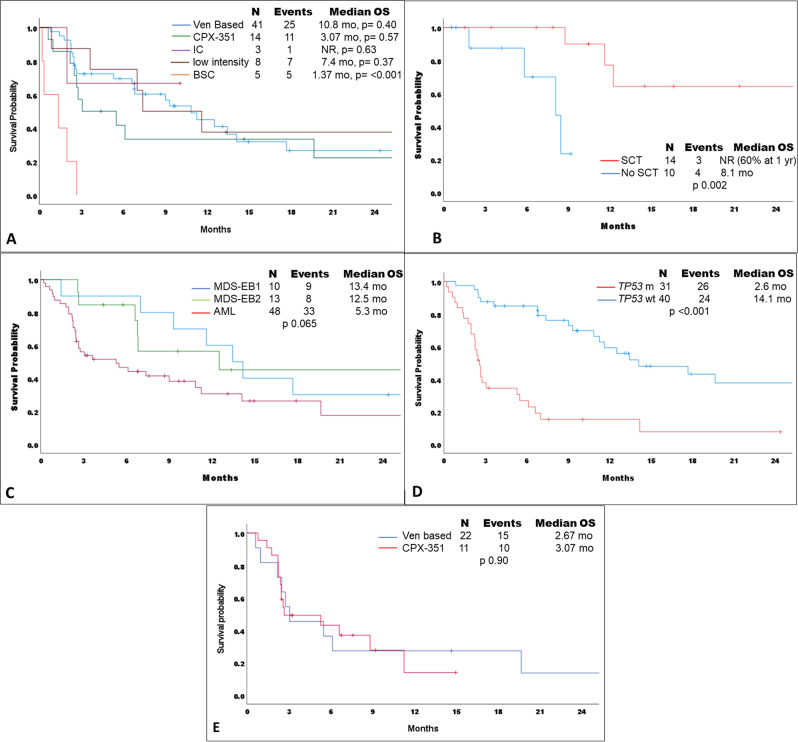
Survival outcome of patients with myelodysplastic syndrome (MDS) progressing on hypomethylating agents (HMA). (**A**) Kaplan Meier survival curve after HMA progression based on different therapies, (**B**) in patients with complete remission (CR) receiving stem cell transplantation (SCT) vs patient in CR and no SCT, (**C**) in MDS-EB1, MDS-EB2 and AML (**D**) in TP53 mutated patients (**E**) in patients with *TP53* mutated and/or complex cytogenetics receiving HMA/low dose cytarabine (LDAC) plus venetoclax versus CPX-351.

Median OS after progressing on HMA across the entire cohort was 7.4 months (95% CI: 2.90–11.89). After excluding 5 patients who did not receive further therapy after HMA progression, the median OS was 9.3 months (95% CI: 4.65–14.0). The median OS after HMA progression in months based on therapy was 10.87 (95% CI; 7.56–14.17, *p* = 0.40), 3.07 (95% CI; 0.00–7.69, *p* = 0.57), 7.40 (95% CI: 0.98–13.81, *p* = 0.37), 1.37 (95%CI: 0.00–3.60, *p* = <0.001), and not reached (NR) (95% CI; not evaluable [NE]-NE, *p* = 0.63) with venetoclax based, CPX-351, other low intensity chemotherapy regimen, best supportive care, and intensive chemotherapy, respectively (Fig. 1A). The median OS in subset of patients, who achieved complete response and received alloHCT was not reached (NR) vs. 8.1 months (95% CI: NE-NE, *p* = 0.02) in the non-alloHCT group (Fig. 1B). The median OS in patient with MDS-EB1, MDS-EB2 and AML after progressing on HMA was 13.4 (95% CI: 9.48–17.45), 12.5 (95% CI: 0.0–36.57) and 5.3 (95% CI: 1.22–9.37) months, respectively (*p* = 0.06 [Fig. 1C]). Among patients with *TP53*m, median OS was 2.60 (95% CI: 2.17–3.02) vs. 14.13 months in *TP53* wild type (95%CI: 6.85–21.40) (*p* = <0.001 [Fig. 1D]). Among patients with *TP53* mutated and/or complex cytogenetics, there was no difference in survival in patients who received HMA/LDAC with venetoclax and CPX-351 therapy (2.67 vs. 3.07, *p* = 0.90) (Fig. 1E).

We performed multivariate analysis using variables which showed significance or trends towards significance in univariate analysis for OS. Achievement of CR/CRi retained significance for improved OS in multivariate analysis (HR: 0.15, 95% CI: 1.17–5.58, *p* = 0.01) whereas *TP53* mutation (HR: 5.18, 95% CI:1.92–13.95, *p* = 0.001), and diagnosis of AML at HMA progression (HR: 2.55, 95% CI: 1.17–5.58, *p* = 0.01) retained significance for inferior OS in multivariate analysis (HR: 3.08, 95% CI:1.42–6.69, *p* = 0.004). Despite demonstrating favorable significance for OS in univariate analysis in CR/CRi patients, alloHCT did not retained significance for better OS in multivariate analysis (HR: 0.61, 95%CI: 0.20–1.82, *p* = 0.38) (Supplementary Table 3).

## Discussion

We presented our experience on outcome of patients with MDS progressing on HMA therapy in the current era. The clinical outcome of patients has improved modestly compared to historical data [3] and patients who achieved complete remission, retained significance for better OS in multivariate analysis.

Azacitdine plus venetoclax based therapy has been a paradigm shift in the management of patients with AML who are ineligible for intensive therapies [6]. Moreover, a recently conducted phase I study, also demonstrated promising efficacy and durable response of the azacitidine plus venetoclax combination in patients with high risk MDS [9]. In our cohort, a venetoclax-based regimen were associated with better OS after HMA progression, however, it did not retain significance in multivariate analysis, most likely due to the heterogenous patient population with a higher proportion of patients harboring *TP53* mutation, having t-MDS, which is distinct from patients in clinical trials. Similar, to what has been reported in the randomized study, we did not observe significant benefit of CPX-351 in achieving remission or improving OS in this group of patients [5].

AlloHCT is the only potential curative option for patients with high risk MDS and secondary AML. In our study patient who achieved remission and underwent alloHCT had significantly better OS, however it did not retain significance for better OS in multivariate analysis. *TP53* mutated high risk MDS/AML are considered high risk disease with poor response to therapy and dismal outcome [10]. Similarly, in our analysis in the era of novel therapies, *TP53* mutation retained poor prognostic significance with a dismal median OS of 2.6 months after progressing on HMA.

We report our institutional experience of clinical outcome of MDS patients progressing after HMA in the era of novel therapies. Response rate and survival outcome are still modest, effective therapies are needed to improve outcome of these high-risk patients.

## Supplementary information

Supplemental Table 1

Supplementary Table 2.

Supplementary Table 3

Supplementary Figure 1

## Data Availability

The data that support the findings of this study are available from the corresponding author upon reasonable request.
