# Supplementary material for: Clinical outcome of myelodysplastic syndrome progressing on hypomethylating agents with evolving frontline therapies: continued challenges and unmet needs
Source: Blood Cancer J. 2022 Jun 24;12(6):93. doi: 10.1038/s41408-022-00691-9 (PMC9232594; doi:10.1038/s41408-022-00691-9)
Supplement: Supplementary file 1 — Supplemental Table 1 [file 41408_2022_691_MOESM1_ESM.docx]

| **Supplemental Table 1. Baseline characteristics, disease status at HMA progression and treatment received by patients with CR/CRi (N=24) undergoing alloHCT vs no alloHCT** | | | |
| --- | --- | --- | --- |
| **Variables** | **AlloHCT**  **(N= 14)** | **No AlloHCT**  **(N=10)** | **P value** |
| Age ≥ 70 yrs | 1 (7) | 6 (60) | 0.02 |
| t-MDS | 3 (21) | 5 (50) | 0.40 |
| High risk CG | 7 (50) | 9 (90) | 0.24 |
| **Disease status at progression** | | | |
| MDS-EB1 | 0 | 2 (20) | 0.20 |
| MDS-EB2 | 3 (21) | 2 (20) | >0.99 |
| AML | 11 (79) | 9 (90) | >0.99 |
| **Mutations at HMA Progression** | | | |
| *D TP53* | 2 (14) | 5 (50) | 0.19 |
| *ASXL1* | 3 (21) | 2 (20) | >0.99 |
| *RUNX1* | 4 (28) | 1 (10) | 0.34 |
| *RAS* | 0 | 1 (10) | 0.46 |
| *TET2* | 2 (14) | 3 (30) | 0.63 |
| *SRSF2* | 2 (14) | 1 (10) | >0.99 |
| *BCOR* | 2 (14) | 1 (10) | >0.99 |
| *IDH1 or IDH2* | 1 (7) | 0 | >0.99 |
| *U2AF1* | 1 (7) | 1 (10) | >0.99 |
| *DNMT3A* | 0 | 1 (10) | 0.44 |
| *STAG2* | 1 (7) | 1 (10) | >0.99 |
| *KDM6A* | 1 (7) | 0 | >0.99 |
| *EZH2* | 0 | 1 (10) | 0.46 |
| *SETB1* | 1 (7) | 0 | >0.99 |
| *GATA2* | 1 (7) | 0 | >0.99 |
| **First-line therapy after progression on HMA** | | | |
| **Venetoclax-based therapy (combination with LDAC or HMA)**  **CPX-351**  **Other low intensity therapies***  **Intensive chemotherapy (7 + 3 or High dose cytarabine based)** | 10 (71)  4 (28)  1 (7)  0 | 9 (90)  0  2 (20)  2 (20) | >0.99  >0.99  0.58  0.20 |
| MDS-EB; MDS with excess blast, LDAC; low dose cytarabine, HMA; hypomethylating agent.  alloHCT; allogeneic stem cell transplant.  *Other low intensity chemotherapy (IDH1/IDH2 inhibitor, Gemtuzumab ozogamicin, ruxolitinib, alternate hypomethylating agent) | | | |
