# Supplementary material for: Clinical outcome of myelodysplastic syndrome progressing on hypomethylating agents with evolving frontline therapies: continued challenges and unmet needs
Source: Blood Cancer J. 2022 Jun 24;12(6):93. doi: 10.1038/s41408-022-00691-9 (PMC9232594; doi:10.1038/s41408-022-00691-9)
Supplement: Supplementary file 2 — Supplementary Table 2. [file 41408_2022_691_MOESM2_ESM.docx]

| **Supplementary Table 2. Predictors of complete response (N= 71)** | | | | |
| --- | --- | --- | --- | --- |
| Variables | Overall | Complete response | No complete response | P value |
| Age ≥ 70 yrs | 25 (35%) | 7 (28%) | 18 (72%) | 0.60 |
| Gender (Male) | 44 (62%) | 14 (32%) | 30 (68%) | 0.79 |
| Therapy-related MDS | 17 (24%) | 7 (41%) | 10 (59%) | 0.55 |
| Complex cytogenetics | 42 (59%) | 10 (24%) | 32 (76%) | **0.04** |
| High risk cytogenetics | 48 (68%) | 14 (29%) | 34 (71%) | 0.28 |
| MDS-EB1 | 10 (14%) | 4 (40%) | 6 (60%) | 0.72 |
| MDS-EB2 | 13 (18%) | 5 (38%) | 8 (62%) | 0.75 |
| AML | 48 (68%) | 15 (31%) | 33 (69%) | 0.59 |
| **Mutations at HMA Progression** | |  |  |  |
| *TP53* | 31 (44%) | 6 (19%) | 25 (81%) | **0.04** |
| *ASXL1* | 15 (21%) | 6 (40%) | 9 (60%) | 0.55 |
| *RUNX1* | 10 (14%) | 4 (40%) | 6 (60%) | 0.71 |
| *RAS* | 9 (13%) | 1 (11%) | 8 (89%) | 0.15 |
| *TET2* | 8 (11%) | 6 (75%) | 2 (25%) | **0.01** |
| *SRSF2* | 5 (7%) | 3 (60%) | 2 (40%) | 0.32 |
| *BCOR* | 5 (7%) | 2 (40%) | 3 (60%) | >0.99 |
| *IDH1 or IDH2* | 4 (6%) | 2 (50%) | 2 (50%) | 0.59 |
| *U2AF1* | 4 (6%) | 3 (75%) | 1 (25%) | 0.10 |
| *EZH2* | 4 (6%) | 1 (25%) | 3 (75%) | >0.99 |
| *CBL* | 3 (4%) | 0 | 3 (100%) | 0.54 |
| *DNMT3A* | 2 (3%) | 2 (100%) | 0 | 0.10 |
| *STAG2* | 2 (3%) | 2 (100%) | 0 | 0.11 |
| *KDM6A* | 2 (3%) | 0 | 2 (100%) | 0.54 |
| *DDX41* | 1 (1%) | 0 | 1 (100%) | >0.99 |
| *SETB1* | 1 (1%) | 1 (100%) | 0 | 0.33 |
| *GATA2* | 1 (1%) | 1 (100%) | 0 | 0.33 |
| **First-line therapy after progression on HMA** | |  |  |  |
| Venetoclax based therapy  CPX-351  Intensive chemotherapy (3+7 or HDAC based)  Other low intensity chemotherapy*  Best supportive care | 41 (58%)  14 (20%)  3 (4%)  8 (11%)  5 (7%) | 19 (46%)  2 (14%)  2 (67%)  1 (12.5%)  0 | 22 (54%)  12 (86%)  1 (33%)  7 (87.5%)  5 (100%) | 0.053 |
| Complete response; CR +CRi+ marrow CR, MDS-EB1; myelodysplastic syndrome with excess blast 1, MDS-EB2; myelodysplastic syndrome with excess blast 2, HMA; hypomethylating agent.  * Other low intensity chemotherapy (IDH1/IDH2 inhibitor, Gemtuzumab ozogamicin, ruxolitinib, alternate hypomethylating agent) | | | | |
