# Supplementary material for: Clinical outcome of myelodysplastic syndrome progressing on hypomethylating agents with evolving frontline therapies: continued challenges and unmet needs
Source: Blood Cancer J. 2022 Jun 24;12(6):93. doi: 10.1038/s41408-022-00691-9 (PMC9232594; doi:10.1038/s41408-022-00691-9)
Supplement: Supplementary file 3 — Supplementary Table 3 [file 41408_2022_691_MOESM3_ESM.docx]

| Supplementary Table 3. Univariate and multivariate analysis for overall survival after HMA progression | | | | | | |
| --- | --- | --- | --- | --- | --- | --- |
| Variable | Univariate Analysis for OS | | | Multivariate Analysis for OS | | |
|  | Median  (months) | 95% Confidence Interval | p-value | Hazard ratio | 95% Confidence Interval | p-value |
| Age ≥ 70 years | 6.63 | 4.46-8.79 | 0.33 |  | | |
| Therapy related-MDS | 5.3 | 0.48-10.11 | 0.16 |  | | |
| Complex cytogenetics | 2.67 | 1.31-4.02 | <0.001 | 1.01 | 0.42-2.38 | 0.97 |
| *TP53* mutated | 2.60 | 2.17-3.02 | <0.001 | 5.18 | 1.92-13.95 | 0.001 |
| *ASXL1* mutated | 9.33 | 4.89-13.76 | 0.59 |  | | |
| *RAS* mutated | 6.80 | 0.95-17.68 | 0.15 |  | | |
| MDS-EB1 | 13.47 | 9.48-17.45 | 0.14 |  | | |
| MDS EB2 | 12.5 | 1.2-36.5 | 0.22 |  | | |
| AML | 5.30 | 1.22-9.37 | 0.02 | 2.55 | 1.17-5.58 | 0.01 |
| **Therapy after HMA progression** |  | | |  | | |
| Venetoclax based regimen | 10.87 | 7.09-14.64 | 0.40 |  | | |
| CPX-351 | 3.07 | 0.00-7.69 | 0.57 |  | | |
| Intensive chemotherapy (3+7 or HDAC based) | NR | (NE-NE) | 0.63 |  | | |
| Other low intensity chemotherapy regimen | 7.40 | 0.98-13.81 | 0.37 |  | | |
| Best supportive care | 1.37 | 0.00-3.60 | <0.001 | 2.39 | 0.84-6.82 | 0.10 |
| Patients with CR/CRi | 35.27 | (NE-NE) | <0.001 | 0.15 | 0.05-0.42 | <0.001 |
| AlloHCT* | NR (62% alive at 12 months) | (NE-NE) | 0.02 | 0.61 | 0.20-1.82 | 0.38 |
| MDS-EB; MDS with excess blast, HMA; hypomethylating agent, HDAC; high dose cytarabine, AlloHCT; allogeneic hematopoietic stem cell transplant, CRi; complete remission with incomplete count recovery, NR; not reached, NE; not evaluable  *Overall survival from the time of achieving complete response and receiving alloHCT | | | | | | |
