# Supplementary figures and images for: Clinical outcome of myelodysplastic syndrome progressing on hypomethylating agents with evolving frontline therapies: continued challenges and unmet needs
Source: Blood Cancer J. 2022 Jun 24;12(6):93. doi: 10.1038/s41408-022-00691-9 (PMC9232594; doi:10.1038/s41408-022-00691-9)

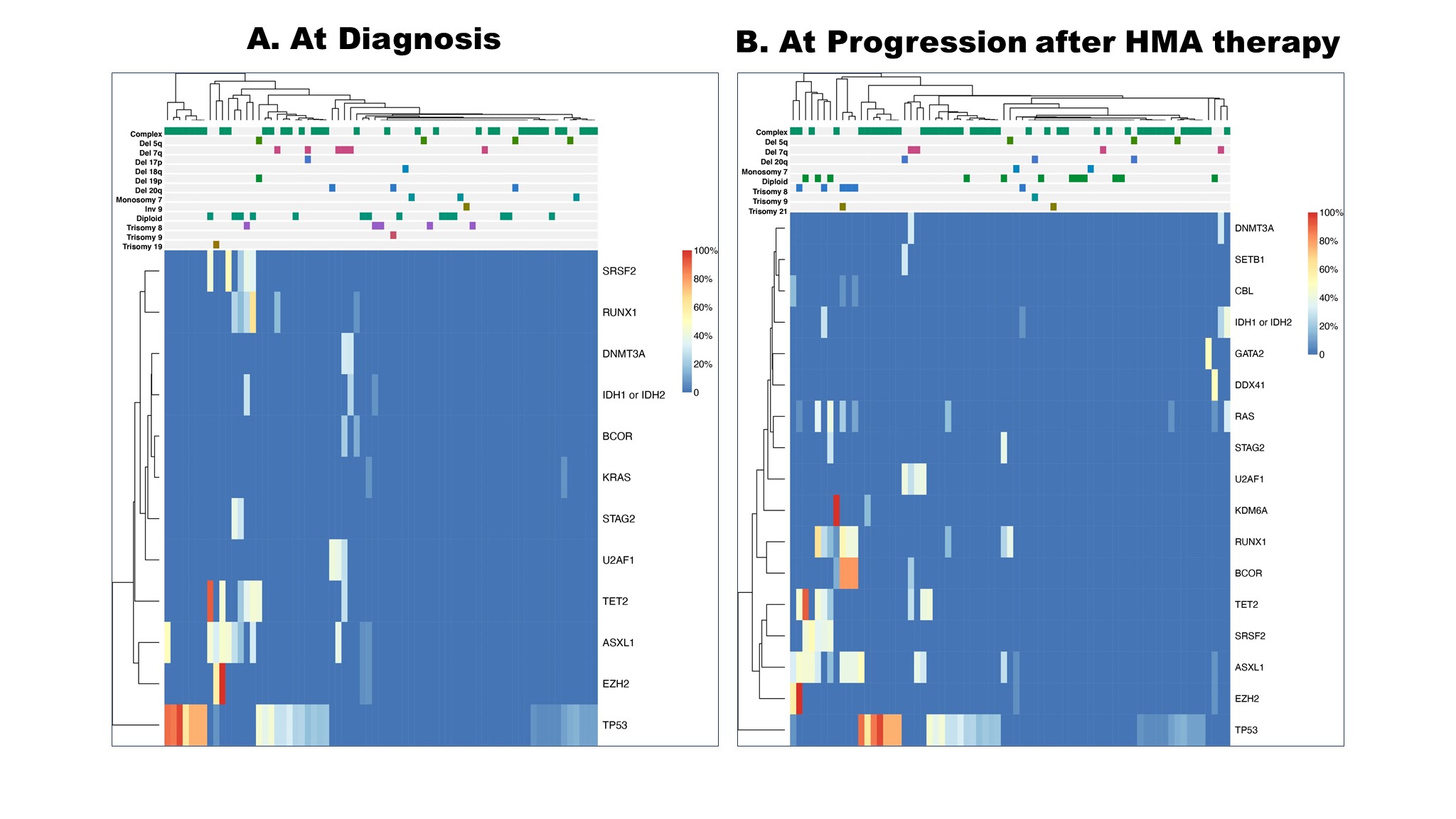

Supplement: Supplementary file 4 — Supplementary Figure 1 [file 41408_2022_691_MOESM4_ESM.jpg]
